# Real-world post-deployment performance of a novel machine learning-based digital health technology for skin lesion assessment and suggestions for post-market surveillance

**DOI:** 10.3389/fmed.2023.1264846

**Published:** 2023-10-31

**Authors:** Lucy Thomas, Chris Hyde, Dan Mullarkey, Jack Greenhalgh, Dilraj Kalsi, Justin Ko

**Affiliations:** ^1^Chelsea and Westminster Hospital NHS Foundation Trust, London, United Kingdom; ^2^Exeter Test Group, Department of Health and Community Sciences, University of Exeter Medical School, Exeter, United Kingdom; ^3^Skin Analytics Ltd., London, United Kingdom; ^4^Department of Dermatology, Stanford Medicine, Stanford, CA, United States

**Keywords:** artificial intelligence, skin cancer, AI for skin cancer, AI as a medical device, DERM, deep ensemble for the recognition of malignancy, Skin Analytics

## Abstract

**Introduction:**

Deep Ensemble for Recognition of Malignancy (DERM) is an artificial intelligence as a medical device (AIaMD) tool for skin lesion assessment.

**Methods:**

We report prospective real-world performance from its deployment within skin cancer pathways at two National Health Service hospitals (UK) between July 2021 and October 2022.

**Results:**

A total of 14,500 cases were seen, including patients 18–100 years old with Fitzpatrick skin types I–VI represented. Based on 8,571 lesions assessed by DERM with confirmed outcomes, versions A and B demonstrated very high sensitivity for detecting melanoma (95.0–100.0%) or malignancy (96.0–100.0%). Benign lesion specificity was 40.7–49.4% (DERM-vA) and 70.1–73.4% (DERM-vB). DERM identified 15.0–31.0% of cases as eligible for discharge.

**Discussion:**

We show DERM performance in-line with sensitivity targets and pre-marketing authorisation research, and it reduced the caseload for hospital specialists in two pathways. Based on our experience we offer suggestions on key elements of post-market surveillance for AIaMDs.

## Introduction

One in every three cancers diagnosed is skin cancer (1). Melanoma is responsible for 90% of skin cancer deaths despite accounting for only ^∼^1% of skin cancers (2). In the United Kingdom (UK), suspected cancer cases are referred to the urgent 2-week-wait (2WW) pathway, in which guidelines suggest that the patient should be seen by a specialist within 2 weeks. Setting this target has been shown to improve the average 5-year melanoma survival by 20%, when compared to historical data (3); however UK cancer registry data shows that the number of 2WW referrals for skin cancer has increased by more than 200% over the last decade, from 159,430 patients in 2009/2010 to 506,456 patients in 2019/2020 (4), leading to significant access pressures and challenges to achieve standards for timely assessment. Adding to the challenge, approximately 25% of melanoma are found in routine (non-urgent) dermatology referrals or follow-up appointments (5). While in 2009/2010, >94% of patients referred for routine dermatology assessment were seen within the target of 18 weeks, only 80% were seen within this target in 2019/20. Increased patient backlogs since the COVID-19 pandemic mean waiting times have increased with routine clinics often cancelled in order to accommodate additional 2WW activity, leading to downstream delays in the skin cancers, including melanomas, presenting in the routine pathway (6). The increase in skin cancer referrals is expected to continue to rise in the coming decades across Europe and the USA due to ageing populations (7).

**FIGURE 1:**
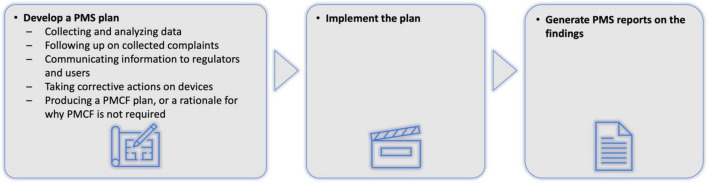
Current post-market surveillance requirements for AIaMDs (11, 12). AIaMD, artificial intelligence as a medical device; PMS, post-market surveillance; PMCF, post-market clinical follow-up.

Artificial intelligence as a medical device (AIaMD) has the potential to help increase workflow efficiency through triage and supporting clinical decisions in skin cancer pathways (8–13); however, evidence for AIaMDs has largely reflected performance using retrospective data (13–16). There remains the need to understand how appropriately regulated AIaMD platforms perform in real-world clinical settings, including how algorithmic improvements or optimisation for different patient populations affects performance over time. Implementing AI systems in real-world settings reveal often-unforeseen complexities (17). Post-market surveillance (PMS) of medical devices, including AIaMDs, is mandated by regulatory agencies, including the UK Medicines and Healthcare Regulatory Agency (MHRA) and the United States Food and Drug Administration (FDA), but these bodies do not stipulate specific approaches on what data should be collected with what frequency, how it should be analysed, or what auditing and quality control processes should take place (Figure 1) (18–20).

Deep Ensemble for Recognition of Malignancy (DERM; Skin Analytics, London, UK) is an AIaMD that uses deep learning techniques to assess dermoscopic images of skin lesions, identify features associated with malignancies and support referral decisions for patients ≥18 years (8–13). DERM is intended to be used for the screening, triage, and assessment of skin lesions, and outputs a suggested diagnosis and referral recommendation. DERM can output a suggested diagnosis of melanoma, squamous cell carcinoma (SCC), basal cell carcinoma (BCC), intraepidermal carcinoma (IEC), actinic keratosis, atypical naevus, or benign, alongside a referral recommendation as agreed for the pathway with local clinical teams. In June 2022 it became the first and only AIaMD for dermatology to be certified as a Class IIa UKCA medical device after an in-depth assessment of Skin Analytics’ quality management system and technical documentation by a UK approved body (SGS United Kingdom Ltd, Leicester, UK) designated by the MHRA (previously DERM was a Class I CE device). This manuscript describes the real-world deployment of DERM in clinical practice at two National Health Service (NHS) Trusts in the UK and proposes an approach for the prospective collection and presentation of real-world PMS data from AIaMDs deployed within clinical pathways for ongoing post-deployment monitoring and quality control.

## Materials and methods

### Study type and location

The analysis is part of the ongoing PMS protocol for DERM to assess its performance in the identification of malignant skin lesions. The data was collected for the service evaluation of DERM commercial deployments in line with its approved intended use. Consistent with medical device regulations, the analysis did not require additional institutional ethics committee approval. All data were collected and analysed according to good clinical practice guidelines and the relevant national laws. All participating patients provided informed consent for their assessment using DERM as part of the service provided by Skin Analytics (data used for case-level analysis), and nearly all (96.7%) provided additional written informed consent for their data to be used for purposes of research and education (data used in the lesion-level analysis).

The data were collected from commercial deployments at University Hospitals Birmingham NHS Foundation Trust (UHB) and West Suffolk NHS Foundation Trust (WSFT). UHB is a large Trust in England treating over 2.8 million patients each year (21). WSFT serves a smaller and predominantly rural geographical area with a population of around 280,000 (22).

### DERM software deployment

During the time covered by the analysis, there were two versions of DERM deployed and we refer to them as DERM-version A (DERM-vA) (July 2021 to April 2022), and version B (DERM-vB) (April 2022 to October 2022). Each version used fixed sensitivity thresholds in order to meet sensitivity targets of at least 95% for melanoma and squamous cell carcinoma (SCC) and 90% for basal cell carcinoma (BCC), intraepidermal carcinoma (IEC) and actinic keratosis. The decision to update to DERM-vB was based on confidence in the revised version’s ability to maintain target threshold sensitivity for malignancy diagnoses while increasing specificity for benign lesions.

### Urgent skin cancer referral pathway

#### Patient selection for DERM deployment

**FIGURE 2:**
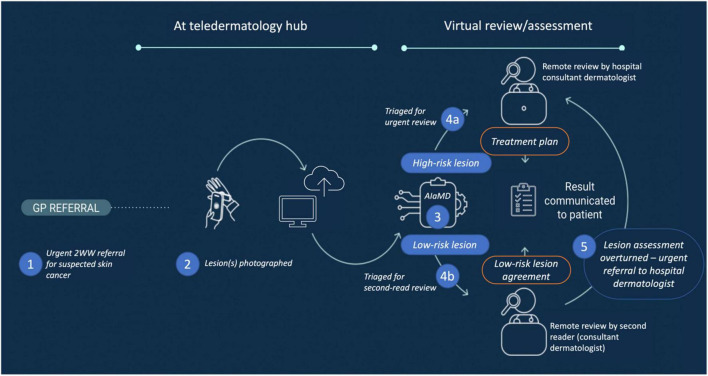
Post-referral pathway for DERM. 2WW, 2-week-wait; AIaMD, artificial intelligence as a medical device; DERM, deep ensemble for recognition of malignancy.

Figure 2 shows the deployment workflow at UHB and WSFT where DERM was used as a triage tool within the urgent 2WW referral pathway. The referral pathways incorporating DERM were designed in collaboration with the clinical teams at both hospitals and consistent with regulated intended use. Patients with suspicious skin lesions were referred by their general practitioner (GP) to attend a teledermatology hub where a clinical photographer or healthcare assistant (CP/HCA) captured standardised photographic images of their lesion(s) and recorded their medical history. Fitzpatrick skin type was optionally assessed and recorded by the CPs/HCAs in conjunction with the patient (23). The imaging team members were also responsible for recording patient consent and assessing whether the patient’s lesions were suitable for assessment by DERM according to its intended use (Table 1).

**TABLE 1:** Eligibility criteria for assessment by DERM according to its intended use.

Inclusions	Exclusions
Lesions are eligible to be assessed by DERM if they are: ∙ Located on adults =18 years ∙ Between 1 and 3 suspicious lesions which are not larger than the dermatoscopic lens (=15 mm)	∙ Patients <18 years ∙ Skin lesions that are not potentially malignant (e.g., rashes, eczema, infectious diseases, lupus) ∙ Skin lesions requiring monitoring for treatment response ∙ Skin lesions that require staging of disease ∙ Non-dermoscopic images of skin lesions ∙ Open ulcerated skin lesions ∙ Skin lesions too large to be entirely imaged within the dermoscopic device (=15 mm) ∙ Lesions obscured by hair, tattoos or scars ∙ Lesions which are subungual, or on mucosal, genital or palmoplantar surfaces ∙ Lesions that have been previously biopsied

DERM, deep ensemble for recognition of malignancy.

#### Lesion imaging

Patients had locating, macroscopic and dermoscopic digital images of their lesion(s) captured by CPs/HCAs using a smartphone (iPhone 6S or 11; Apple, CA, USA) and polarised dermoscopic lens attachment (Dermlite DL1 basic, Schuco, UK). For some patients, additional images were captured using a digital single-lens reflex camera (DSLR) with a dermoscopic lens attachment for clinical use (the DSLR images were not assessed by DERM). Routine post-market auditing identified that a small number of images were captured in error using an unapproved non-polarised dermoscopy tool; however, none of these were excluded from this analysis.

#### DERM assessment and triage recommendation

The dermoscopic image of eligible patients’ lesion(s) was assessed by DERM, which provided a suggested diagnosis and corresponding recommendation, e.g., discharge from pathway or refer to the hospital-based consultant dermatologist for review. DERM classified lesions as melanoma, SCC, BCC, IEC, actinic keratosis, atypical naevus, or benign (six subcategories of benign lesions were grouped together aligned with patient management). DERM’s output regarding suggested diagnosis corresponded to the highest risk possibility rather than the most likely classification, e.g., if a lesion was more likely to be a seborrhoeic keratosis but also crossed the defined threshold for melanoma, DERM would output melanoma. Patients for whom all lesions were assessed by DERM and classified as benign were eligible for discharge. Patients with any lesion classified by DERM as not benign or excluded from DERM assessment remained on the urgent 2WW pathway.

#### Human in the loop: second-read review for benign lesions

Although not required given the Class IIa medical device designation, a second-read review of all cases marked for discharge by DERM was conducted within 48 h by a consultant dermatologist, listed on the UK General Medical Council’s Specialist Register (second-read reviewer), working with Skin Analytics and who could agree with or overturn the recommendation to discharge from the 2WW skin cancer pathway. The second-read reviewer had access to the patient’s clinical information and smartphone-captured images but not the DSLR images. If the second-read reviewer overturned the recommendation to discharge, the case was referred for hospital dermatologist review.

Cases marked for urgent referral directly by DERM, indirectly via the second-read review, or excluded from DERM assessment were assessed virtually by a hospital consultant dermatologist to provide a clinical diagnosis and final recommendation, e.g., discharge, surgery/biopsy, or clinical follow-up. All hospital dermatologists had access to the patient’s clinical information, smartphone images, and additional DSLR images (if available).

### Lesion- and case-level analysis

Two different populations were analysed: (1) DERM-assessed lesions that had a final diagnosis (defined by histology for malignant lesions and by dermatologist clinical assessment or histology if available for non-malignant lesions) and the patient had provided additional research consent allowing for assessment of performance of DERM on specific lesions; and (2) case-level data gathered from all patients who were assessed within the pathways described above allowing for assessment of performance of the service integrating DERM overall. The latter includes cases with no DERM assessment (e.g., due to exclusions or technical issues) and where the final diagnosis is still pending. The two populations are expected to be sufficiently similar for interpretation of results to be meaningful with a high patient uptake for additional research consent.

#### Performance of DERM lesion classification (lesion-level population)

The performance of DERM was evaluated by comparing its lesion classification and management recommendation with the final diagnosis. The performance of DERM compared to the final diagnosis was analysed as to whether it correctly classified lesions as: (1) melanoma or not, whereby a true positive is a histology-confirmed melanoma labelled melanoma by DERM; (2) malignancy or not, whereby a true positive is a histology-confirmed melanoma, SCC, BCC or rare skin cancer labelled as melanoma, SCC or BCC by DERM; and (3) refer or not, whereby a true positive is a histology-confirmed melanoma, SCC, BCC or rare skin cancer or a histology/clinically confirmed Bowen’s disease, actinic keratosis, atypical naevus or other premalignant lesion labelled as anything other than benign by DERM (Supplementary Table 1). Sensitivity, specificity, negative predictive value (NPV), positive predictive value (PPV), and number needed to biopsy/refer/treat (NNB) with their 95% confidence intervals were calculated for all three levels of lesion classification.

#### Performance of service (case-level population)

The 2WW skin cancer pathway involving DERM was assessed in terms of the proportion of patients with lesions who were safely discharged after DERM, second-read review and hospital dermatologist assessment, respectively. Cancers confirmed from DERM-discharged cases overturned by the second-read and instances where lesions were discharged but histologically confirmed as a cancer on a subsequent presentation (“repeat presentations”) were identified and underwent a root cause analysis including a panel review (three dermatologists and an AI expert). Sensitivity for the overall service is reported, whereby repeat presentations of lesions occurring within 6 months of initial discharge are considered false negatives.

### Proposal for monitoring post-market surveillance

Based on this experience of deploying an AIaMD in real-world clinical practice, the authors present the current, as well as proposed framework for post-deployment monitoring and quality control of AI in real-world clinical settings.

## Results

### Patient population

**FIGURE 3:**
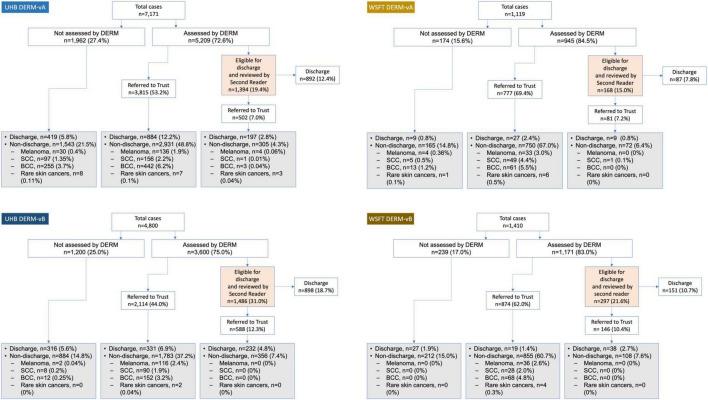
Post-deployment DERM referral pathway at two secondary care hospitals in England, United Kingdom (case-level population). Lesions that were not assessed by DERM were referred straight to hospital teledermatology review. Grey- and orange-shaded boxes indicate hospital teledermatology or second-reader review, respectively. For non-discharged lesions, details are provided only for lesions with a skin cancer diagnosis. BCC, basal cell carcinoma; DERM-vA or -vB, deep ensemble for recognition of malignancy version A or B; SCC, squamous cell carcinoma.

In total, 8,809 cases (patients) at UHB and 2,116 cases at WSFT were assessed by DERM (case-level population; Figure 3). The number of lesions with a final diagnosis and patient consent for research was 7,220 at UHB and 1,351 at WSFT (lesion-level population). A broad age range of patients were included (18–100 years) and all Fitzpatrick skin types were represented with the majority being skin types I–IV (Table 2), reflecting skin cancer incidence among these populations (24).

**TABLE 2:** Skin type and age of participants (lesion-level population*).

	UHB DERM-vA (*n* = 4,635)	WSFT DERM-vA (*n* = 709)	UHB DERM-vB (*n* = 2,585)	WSFT DERM-vB (*n* = 642)
**Skin type**				
Fitzpatrick type I	248 (5.4%)	125 (17.6%)	215 (8.3%)	74 (11.5%)
Fitzpatrick type II	721 (15.6%)	425 (59.9%)	656 (25.4%)	345 (53.7%)
Fitzpatrick type III	607 (13.1%)	149 (21%)	619 (23.9%)	205 (31.9%)
Fitzpatrick type IV	127 (2.7%)	7 (1%)	132 (5.1%)	14 (2.2%)
Fitzpatrick type V	25 (0.5%)	1 (0.1%)	46 (1.8%)	4 (0.6%)
Fitzpatrick type VI	3 (0.1%)	1 (0.1%)	14 (0.5%)	0 (0%)
Not recorded	2904 (62.7%)	1 (0.1%)	903 (34.9%)	0 (0%)
**Age range, years**				
18–29	393 (8.5%)	43 (6.1%)	247 (9.6%)	41 (6.4%)
30–39	502 (10.8%)	56 (7.9%)	320 (12.4%)	61 (9.5%)
40–49	505 (10.9%)	60 (8.5%)	302 (11.7%)	71 (11.1%)
50–59	805 (17.4%)	106 (15%)	461 (17.8%)	116 (18.1%)
60–69	874 (18.9%)	123 (17.3%)	503 (19.5%)	129 (20.1%)
70–79	983 (21.2%)	192 (27.1%)	458 (17.7%)	147 (22.9%)
=80	573 (12.4%)	129 (18.2%)	294 (11.4%)	77 (12%)
Not recorded	0 (0%)	0 (0%)	0 (0%)	0 (0%)

Data are presented as n (%). *Lesions were included in the analysis if there was a confirmed final diagnosis (histology for malignant lesions and dermatologist opinion or histology for non-malignant lesions). Lesions were excluded from the analysis if they did not fulfil the inclusion criteria for lesion assessment by DERM, were not analysed by DERM for any technical reason or were pending final diagnosis defined by histology for malignant lesions and by dermatologist clinical assessment or histology if available for non-malignant lesions. DERM, deep ensemble for recognition of malignancy.

### Performance of DERM lesion classification (lesion-level population)

Post-deployment performance of DERM-vA and DERM-vB are reported in Table 3. Both versions of DERM performed with very high levels of sensitivity for skin cancer detection (96.0–100.0%). DERM-vB labelled 246 out of 248 lesions as skin cancer; the remaining two lesions were referred with a label of Bowen’s disease and later confirmed to be BCC. Specificity was 40.7–49.4% for DERM-vA and 70.1–73.4% for DERM-vB. A total of 159 lesions were assessed in patients with Fitzpatrick skin types V and VI, for which 94 lesions had a final diagnosis, including BCC (*n* = 1) and IEC (*n* = 1), and actinic keratosis (*n* = 1), all correctly referred by DERM, and atypical naevus (*n* = 3) pending face-to-face assessment, and the remainder were benign with a benign specificity of 44.3% (39/88).

**TABLE 3:** Post-deployment performance of DERM (lesion-level population).

Lesions, % (n/N) [95% confidence interval]	Melanoma or not	Malignant or not	Refer or not
**Sensitivity**			
DERM-vA (UHB)	95.0% (133/140) [90–97.6%]	96.0% (722/752) [94.4–97.2%]	93.4% (1667/1784) [92.2–94.5%]
DERM-vA (WSFT)	97.0% (32/33) [84.7–99.5%]	99.3% (149/150) [96.3–99.9%]	94.9% (316/333) [92–96.8%]
DERM-vB (UHB)	100.0% (58/58) [93.8–100%]	98.9% (178/180) [96–99.7%]	87.4% (673/770) [84.9–89.6%]
DERM-vB (WSFT)	100.0% (18/18) [82.4–100%]	100.0% (68/68) [94.7–100%]	89.5% (222/248) [85.1–92.7%]
**Specificity**			
DERM-vA (UHB)	58.8% (2643/4495) [57.4–60.2%]	45.0% (1747/3883) [43.4–46.6%]	49.4% (1408/2851) [47.6–51.2%]
DERM-vA (WSFT)	63.2% (427/676) [59.5–66.7%]	33.1% (185/559) [29.3–37.1%]	40.7% (153/376) [35.8–45.7%]
DERM-vB (UHB)	80.9% (2045/2527) [79.3–82.4%]	64.8% (1559/2405) [62.9–66.7%]	73.4% (1333/1815) [71.4–75.4%]
DERM-vB (WSFT)	80.4% (502/624) [77.2–83.4%]	60.6% (348/574) [56.6–64.5%]	70.1% (276/394) [65.4–74.4%]
**Negative predictive value**			
DERM-vA (UHB)	99.7% (2643/2650) [99.5–99.9%]	98.3% (1747/1777) [97.6–98.8%]	92.3% (1408/1525) [90.9–93.6%]
DERM-vA (WSFT)	99.8% (427/428) [98.7–100%]	99.5% (185/186) [97–99.9%]	90.0% (153/170) [84.6–93.7%]
DERM-vB (UHB)	100.0% (2045/2045) [99.8–100.0%]	99.9% (1559/1561) [99.5–100.0%]	93.2% (1333/1430) [91.8–94.4%]
DERM-vB (WSFT)	100% (502/502) [99.2–100%]	100% (348/348) [98.9–100.0%]	91.4% (276/302) [87.7–94.1%]
**Positive predictive value**			
DERM-vA (UHB)	6.7% (133/1985) [5.7–7.9%]	25.3% (722/2858) [23.7–26.9%]	53.6% (1667/3110) [51.8–55.3%]
DERM-vA (WSFT)	11.4% (32/281) [8.2–15.6%]	28.5% (149/523) [24.8–32.5%]	58.6% (316/539) [54.4–62.7%]
DERM-vB (UHB)	10.7% (58/540) [8.4–13.6%]	17.4% (178/1024) [15.2–19.8%]	58.3% (673/1155) [55.4–61.1%]
DERM-vB (WSFT)	12.9% (18/140) [8.3–19.4%]	23.1% (68/294) [18.7–28.3%]	65.3% (222/340) [60.1–70.2%]
**Number needed to biopsy, treat or refer**			
DERM-vA (UHB)	14.9 (1985/133) [12.7–17.6]	4 (2858/722) [3.7–4.2]	1.9 (3110/1667) [1.8–1.9]
DERM-vA (WSFT)	8.8 (281/32) [6.4–12.2]	3.5 (523/149) [3.1–4]	1.7 (539/316) [1.6–1.8]
DERM-vB (UHB)	9.3 (540/58) [7.3–11.9]	5.8 (1024/178) [5.0–6.6]	1.7 (1155/673) [1.6–1.8]
DERM-vB (WSFT)	7.8 (140/18) [5.2–12.1]	4.3 (294/68) [3.5–5.4]	1.5 (340/222) [1.4–1.7]

DERM, deep ensemble for recognition of malignancy; UHB, University Hospital Birmingham; WSFT, west sussex foundation trust.

#### Rare skin cancers

Among the lesions assessed, 19 rare skin cancers (defined as not melanoma, SCC or BCC and comprised trichilemmal carcinoma, dermal sarcoma, atypical fibroxanthoma and marginal zone lymphoma) were identified, of which DERM-vA and -vB labelled 13/16 and 3/3 lesions as melanoma or SCC, respectively. Three lesions were labelled “benign” by DERM-vA: two subdermal foci of melanoma with no cutaneous changes [during root cause analysis (RCA) these lesions were assessed as not having been suitable for the service] and one marginal zone lymphoma with cutaneous changes. Complete confusion matrixes for DERM lesion classifications are provided in Supplementary Table 3.

### Performance of service (case-level population)

#### Second-read review

For DERM-vA, 1,393/5,209 cases assessed (26.8%) were labelled as eligible for discharge at UHB. The second-read reviewer overturned 502/1,393 cases (36.0%), of which the hospital dermatologist discharged 197/502 (39.2%). A total of 11 skin cancers (2.0%) were found among these cases. At WSFT, 168/945 (17.8%) cases evaluated by DERM-vA were labelled as eligible for discharge. The second read overturned 81/168 (48.2%) cases, of which the hospital dermatologist discharged 9/81 (11.1%). One skin cancer was found (1.2%) among these cases.

For DERM-vB, 1,486/3,603 cases assessed (41.2%) were labelled as eligible for discharge at UHB. The second read overturned 588/1,486 cases (39.6%), of which the hospital dermatologist discharged 232/588 (39.5%). No skin cancers were found (0% conversion) among lesions marked eligible for discharge by DERM-vB. At WSFT, 297/1,410 cases (25.4%) evaluated by DERM-vB were labelled as eligible for discharge. The second read overturned 146/297 cases (49.2%), of which the hospital dermatologist discharged 38/146 (26.0%). No skin cancers were found (0% conversion).

#### Repeat presentations

No lesions have been assessed by DERM-vA or -vB and discharged from these pathways with a subsequent re-presentation and diagnosis of cancer (service sensitivity 100% to date); however, there have been four lesions that presented twice to the UHB pathway before July 2021, with the second presentation resulting in a histologic diagnosis of skin cancer (melanoma, *n* = 2; BCC, *n* = 2; Supplementary Table 2), though only one (a melanoma) was within 6 months. These were all either triaged by DERM to Trust teledermatology review (*n* = 4) or excluded from assessment by DERM at either the first (*n* = 2) or second presentation (*n* = 2) to the pathway.

## Discussion

Herein, we present a real-world deployment performance evaluation for the AIaMD, DERM, which uses deep learning techniques to assess dermoscopic images of skin lesions for patients who were referred to an urgent skin cancer pathway. During the assessed period, DERM performed at or above the expected level for all malignant and pre-malignant lesion types based on 1,150 confirmed malignancies, including 249 melanomas and 19 rare malignancies. DERM-vB correctly referred all skin cancers in these pathways and had a specificity greater than the previous DERM-vA version. During this period, no patients were discharged from the service and re-presented later with the same lesion being diagnosed as skin cancer. While other published evidence demonstrate a gap in model performance when evaluating real-world prospective clinical use compared with *in silico* data (25–27), our analysis demonstrates that DERM can be deployed safely in live clinical services accessible to patients from a broad range of age groups and skin types, with sensitivity and specificity in-line with target thresholds and performance demonstrated in pre-marketing authorisation studies (8–13).

A critical issue is whether the estimates of performance are valid in this real-world deployment. We examined this by considering the validity and applicability issues identified in the QUADAS-2 tool (Supplementary Appendix A), the most commonly used quality assessment tool for test accuracy studies (28). This reveals that the general openness to bias is similar to many studies included in systematic reviews, particularly those produced by the Cochrane Collaboration. The area of greatest concern is patient selection, whereby there is not a perfectly consecutive series of patients due to current exclusion criteria; however, given that ^∼^80% of all patients referred for suspected skin cancer to UHB and WSFT were seen by these pathways, there is a high level of consecutiveness. Other concerns relate to the information provided by DERM being available to those making the reference standard diagnosis, although arguably this is unlikely to introduce bias because of the current general scepticism about the value of AI by the medical community and the positioning of dermatologists in the pathway to either review lesions already identified as high risk or to actively screen for false negatives. Finally, there is differential verification in the reference standard (ground truth), but this is a near universal problem for the evaluation of the accuracy of skin cancers because it is unethical to biopsy all patients in a study, particularly those deemed as having a low likelihood of cancer and this mirrors limitations within any evaluation of current standards of care. Concerning applicability, the study scores highly, and this should be seen as a particular strength for a real-world deployment.

Although the DERM PMS programme was established before the CLEAR consensus guidelines were published for evaluation of AI studies in dermatology (29), our post-deployment data collection methods align with the relevant checklist items, including prospective data collection, and providing details of image acquisition, patient skin colour, deployment referral pathway, hierarchical outputs, and technical assessments of performance. We did not collect ethnicity or patient sex as we operated on the principle of only collecting data necessary to inform or evaluate DERM performance as part of DERM’s PMS. We plan to re-evaluate the future role of collecting and reporting on these demographic data elements.

Although DERM used images captured using an iPhone camera, it is not a smartphone app *per se*. In contrast, there are numerous smartphone apps intended to classify skin lesions (30). An analysis of 43 such apps showed that these had a mean sensitivity of 0.28 [95% confidence interval (CI) 0.17–0.39], mean specificity of 0.81 (95% CI 0.71–0.91) and mean accuracy of 0.59 (95% CI 0.55–0.62) for the detection of melanoma (31). Direct-to-consumer products do not meet the standards necessary for utilisation in clinical pathways. Direct-to-consumer products generally are not integrated into healthcare services that enable definitive diagnosis, management recommendation and treatment.

Our real-world evidence suggests that DERM can make autonomous decisions to discharge patients with benign skin lesions from the urgent cancer pathway. The second-read reviewer overturned 40–50% of cases that DERM had marked as eligible for discharge; however, for DERM-vA, only 1.2% of these cases resulted in skin cancer diagnosis and with DERM-vB, none resulted in a skin cancer diagnosis. Cost-benefit and economic analyses for the service are ongoing and supported by a 2021 NHS AI in Health and Care Award (32). Adherence to regulatory standards and continuous monitoring need to ensure that autonomous decisions made by AIaMDs are carried out safely while augmenting the non-specialist clinicians’ involvement in care, including in the appropriate counselling of patients.

### Suggestions for post-market surveillance for AI medical devices

Medical device regulations which govern AIaMDs are in place to support access to safe and effective devices and limit access to products that are unsafe. This includes the requirement that manufacturers must submit vigilance reports to the relevant regulatory agency when certain incidents occur involving their device. Although all medical devices require PMS as part of the manufacturer’s obligations to ensure that their device continues to meet appropriate standards of safety and performance for as long as it is in use, these requirements are not specific and there is currently limited transparency on how PMS is being conducted by manufacturers. As such, we recommend that manufacturers monitor and publish real-world evaluations of their AIaMDs within a clinically relevant timeframe. There is a need for PMS alignment to reduce variability of surveillance design and analysis and to improve comparability with other AlaMDs or to monitor the same device over time. Guidelines for best-practice evaluation of image-based AI development in dermatology (CLEAR Derm consensus) provide a checklist to ensure consistency but these are aimed at clinical development as opposed to post-deployment data collection. Nevertheless, many of the items listed in the checklist are pertinent to PMS (29). Manufacturers of AIaMDs may also benefit from specific, tangible advice to support their PMS development plans and regulators and adopters (users) should have a good understanding of what to expect from real-world evidence collected as part of PMS plans (Table 4). PMS processes need to have automatic safeguards or systems in place to ensure rigorous monitoring for robust performance of the AIaMD. Moreover, collecting, analysing, and publishing PMS data requires significant collaboration between the manufacturer, healthcare provider partners, healthcare professionals and patients. Automatic systems, such as electronic patient records that auto-populate a registry database may improve the collection of long-term patient outcomes that go beyond monitoring the specificity and sensitivity of the AIaMD.

**TABLE 4:** Post-market surveillance recommendations for monitoring AIaMDs deployed in real-world settings.

When	Responsibility	Recommendation	Process consideration	Dermatology example
Prior to deployment	Manufacturer	Document and share PMS Plan with healthcare provider partners. This should include final outcome definitions, data sources and cadence of performance reports	Time and resource implications for healthcare providers to acknowledge/review the PMS plan	Agree all skin cancer outcomes will be based on histopathologically confirmed cases to mitigate for inter-clinician variation and mirror clinical practice
	Manufacturer and deploying organisation	Agree how data will be shared with the manufacturer to support	Time and resource implications for healthcare providers. Data privacy and data sharing compliance with patient consent and local laws	Access to histology reports for cases assessed through the service
	Manufacturer	Agree RCA process for false negatives	Process may also be applicable for further investigation of other areas of interest, e.g., low incidence populations, rare diseases, common false positives	Consideration of patient history vs. macro imaging vs. dermoscopic imaging as key factors in cancer diagnosis of a false negative
During deployment and as set out in PMS plan	Manufacturer	Agreement on how many cases should be reviewed initially with second-read review (human-in-the-loop) as a safety net, with a performance review before removal	Time and resource savings should only be considered once the AIaMD has proven to operate within acceptable safety limits	Performance at or above stated target sensitivity for skin cancer over a 6-month+ period at =2 deployment sites
	Manufacturer and/or deploying organisation	Active search for repeat presentations	Will patients always present through the same pathway? If not, does the deploying organisation have better data to search for patients presenting with the same complaint more than once?	Has the same patient presented to the service twice regarding the same lesion?
	Manufacturer	Follow RCA Process for all false negatives and share findings with deploying organisation	Time and financial cost associated with conducting process	Multi-step process including detailed review of histology, review of case by panel of dermatologists, adversarial testing
	Manufacturer	Publish performance report including reference to any available benchmark data (i.e., to allow comparison with other health providers and performance over time; ideally, data would be published in a peer-reviewed journal)	Peer-review publication may introduce delays and so as a minimum the performance should be made available to existing partners or upon request by health organisations considering using the AIaMD	Quarterly Performance Report shared with partners including comparison of new pathway performance vs. nationally available conversion rates
	Manufacturer and/or deploying organisation	Risk-registry database to identify common themes and to investigate if agreed thresholds are breached*	Quickly identify any performance issues and their cause	Ensure correct hardware is in use to collect skin lesion images

AIaMD, artificial intelligence as medical device; PMS, post-market surveillance; RCA, root cause analysis. *This should build on existing quality management system and clinical risk management requirements already mandated for medical device manufacturers.

#### Data management

Post-market surveillance data collection methods need to be planned before AIaMD deployment, including what is needed to ensure ongoing performance and any baseline values that would be useful. The manufacturer needs to put in place plans for auditing and data quality assurance.

A period of continuous monitoring is required to ensure that the AIaMD is performing as expected, especially when there are software updates or changes to the deep learning algorithms that may affect performance. As such, processes need to be able to quickly identify and analyse performance errors so that these can be corrected, and future occurrences prevented (33). For example, during initial deployment, a second-read review would provide a safety net until performance is at or above the expected targets. A statistically significant amount of continuous data with performance at or above expected targets is achieved in alignment with regulatory standards and intended use; for DERM, the demonstration across two distinct locations may support its deployment without a human second-read.

Manufacturers need to start conversations with healthcare providers as early as possible, to consider contractual obligations or incentives to ensure the manufacturer has access to data required for PMS in a timely manner. There is considerable variation in terms of which stakeholder owns or can access the data required in any given organisation. Data requirements need to be agreed with all stakeholders, with ongoing discussions and iterations to ensure the data being collected and analysed remain relevant for performance assessment. Consideration also needs to be given to liability and data privacy issues, including General Data Protection Regulation (GDPR) or equivalent local legislature and the patient’s right to withdraw consent.

#### Root cause analysis for quality control issues, false negative classifications, and “near-misses”

Deep Ensemble for Recognition of Malignancy has now classified more than 60,000 skin lesions in real-world settings across eleven NHS pathways in the UK that have identified 5,385 histology-confirmed malignant lesions (34–36). Specific guidance on AI quality control and improvement in hospitals has been recently published, which describes detection of errors in AI algorithms, monitoring software updates, cause-and-effect analysis for a drop in performance, monitoring changes to input or target, the challenges in monitoring AI system variables, and adapting the FDA’s existing Sentinel Initiative for monitoring AIaMDs after deployment (37).

In terms of reviewing a false negative, case review should be undertaken by a relevant specialist. When a false negative was identified for DERM post-deployment, a root cause analysis was conducted. Histology reports were reviewed for factors such as uncertainty of diagnosis, staging of disease, subtype of disease and perineural and perivascular invasion. A panel of three dermatologists plus an AI expert reviewed all case details including clinical and dermatoscopic images and histology reports, and assessed which factor(s) contributed to the false negative result. Current labels include whether the lesion should have been excluded from DERM assessment or other technical factors, had an unusual presentation or was due to AI performance issues. Any lesion(s) that were a repeat presentation and were confirmed to have cancer were also identified for false negative review and for these cases the panel was asked to comment on whether the malignancy was likely to be present at the point of the first assessment or whether the transformation took place in the interval between appointments. These false negatives should be collated in a “risk” registry and assessed to identify common themes with thresholds for escalation for more in-depth review.

#### Considerations arising from assessment of openness to bias

Our reflection on the validity of our data also suggests ways in which the process of PMS data collection could be optimised to maximise validity. Careful attention to documenting and describing legitimate losses to follow-up, patients who are ineligible for assessment and technical failures is particularly important for the credibility of the information. Moreover, documenting repeat presentations provides reassurance that cancers are not being missed. As such, PMS protocols should clearly describe the time intervals that are being used to confirm that a repeat presentation has not occurred. Clear information about how the AIaMD is being used in the final diagnosis would also be helpful to alert to the possibility of bias if there appears to be heavy reliance on its assessment.

#### Future directions—Looking beyond AIaMD performance at patient outcomes

We are looking to make improvements to the quality of care provided to patients with suspicious skin lesions. Currently, PMS of AIaMDs is focussed on performance, but ultimately data collected as part of PMS should include clinically meaningful metrics, such as reporting the timeliness of diagnosis of malignant lesions after the initial GP referral, time to excision/treatment, provide more information about lesion characteristics (e.g., staging) and importantly longer-term outcomes such as progression-free or overall survival.

### Limitations

Deep Ensemble for Recognition of Malignancy is not intended to provide a definitive diagnosis for skin cancer, as the final diagnosis is confirmed by histopathology or a dermatologist for the case of high-risk lesions. Future opportunities exist to realise further potential of DERM to allow patients with benign lesions to be discharged as quickly as possible, including reducing the exclusion rate (e.g., by using larger dermatoscopic lenses) and using additional data to develop and validate its use on mucosal, palmoplantar and subungual lesions. Human factors and user interaction including explainability could also be assessed in future but was outside the scope of this analysis (38). More explainable outputs could include techniques such as saliency maps, differential diagnosis using conformal predictions, or argumentation approaches (39, 40). However, any additional outputs would need to be validated by human factors and reader studies.

Skin cancers are less common in people with skin of colour (Fitzpatrick skin types V and VI) (24, 41). The current exclusion of palmoplantar and subungual lesions means that DERM cannot be used on the areas where patients with darker skin colour are most likely to develop melanoma (42). Continued surveillance is needed to ensure that patients with darker skin tones have equitable access to the DERM service particularly because patients with darkly pigmented skin often have a more advanced initial melanoma and higher mortality rate than fair-skinned patients (43). This is, however, not a concern that is exclusive to AIaMD–powered skin cancer pathways but rather that appropriate vigilance is required for any skin cancer service.

There is currently a lack of robust baseline operational data from prior to developing and implementing the DERM pathway for UHB and WSFT for number of biopsies, non-melanoma skin cancers diagnosed and pre-malignant diagnoses, or discharge rates for patients with non-malignant lesions. As such, we cannot currently determine how these metrics have changed since the deployment of the DERM pathway.

## Overall conclusion

The real-world implementation of DERM, an AIaMD, in two NHS skin cancer pathways, demonstrates high levels of performance. DERM is accessible to adults of all ages (18–100 years) and has been used to assess potential malignant skin lesions in all Fitzpatrick skin types I–VI. The performance of DERM will continue to be assessed as part of its PMS, including continued consideration of accessibility across the whole population. The performance demonstrated to date provides sufficient evidence to support the removal of the second-read for low-risk lesions in order to maximise health system benefits safely. Based on our experience we offer some suggestions on key elements of post-market surveillance for AIaMDs.

## Data availability statement

The datasets presented in this article are not readily available because the data included in this manuscript have been collected as part of the routine post-market surveillance programme for DERM, conducted by Skin Analytics, London. Requests to access the datasets should be directed to DK, dilraj@skinanalytics.co.uk and DM, dan@skinanalytics.co.uk.

## Ethics statement

Ethical approval was not required for the studies involving humans because this study was conducted as part of ongoing service evaluation of skin cancer pathways and post-market surveillance of a medical device. The studies were conducted in accordance with the local legislation and institutional requirements. The participants provided their written informed consent to participate in this study.

## Author contributions

LT: Conceptualization, Formal Analysis, Investigation, Methodology, Supervision, Writing – review and editing. CH: Conceptualization, Formal Analysis, Investigation, Methodology, Supervision, Writing – review and editing. DM: Conceptualization, Data curation, Formal Analysis, Funding acquisition, Investigation, Methodology, Project administration, Resources, Supervision, Visualization, Writing – original draft, Writing – review and editing. JG: Conceptualization, Data curation, Formal Analysis, Funding acquisition, Investigation, Methodology, Resources, Software, Supervision, Validation, Writing – review and editing. DK: Conceptualization, Data curation, Formal Analysis, Investigation, Methodology, Project administration, Resources, Supervision, Validation, Visualization, Writing – original draft, Writing – review and editing. JK: Conceptualization, Formal Analysis, Investigation, Methodology, Supervision, Writing – review and editing.
