# Supplementary material for: Real-world post-deployment performance of a novel machine learning-based digital health technology for skin lesion assessment and suggestions for post-market surveillance
Source: Front Med (Lausanne). 2023 Oct 31;10:1264846. doi: 10.3389/fmed.2023.1264846 (PMC10645139; doi:10.3389/fmed.2023.1264846)
Supplement: Supplementary file 1 [file Data_Sheet_1.docx]

**Appendix A**

**QUADAS-2 - *Real-world post-deployment data of a novel machine learning-based digital health technology for skin lesion assessment and suggestions for best practice in post-market surveillance***

**Phase 1: State the review question: *Test accuracy of AI for triaging patients referred to hospital for assessment of skin lesions suspicious of malignancy***

| *Patients (setting, intended use of index test, presentation, prior testing):*  Patients referred from primary care with skin lesions suspicious of malignancy. AI system would be used to triage referrals; some patients could be discharged without being seen by the hospital team if the AI system identified them as non-malignant. No prior testing other than GP was concerned that on visual appearance and history of lesion is concerned that the lesion could be malignant |
| --- |
| *Index test(s):*  Artificial Intelligence as a medical device, specifically Deep Ensemble for Recognition of Malignancy (DERM; Skin Analytics, London, UK) |
| *Reference standard and target condition:*  Several possible target conditions   - Melanoma vs non melanoma - Malignancy vs non-malignancy (including pre-malignant conditions) - Conditions requiring referral (malignancy and pre-malignant conditions which may vary depending on local clinical pathways) vs non-referable conditions   Reference standard ideally would be based on biopsy of all lesions irrespective of whether they initially tested positive or not.  This is generally unachievable in practice, so absence of target condition is often based on clinical opinion where biopsy is not taken, confirmed by a period of follow-up during which the patient has not re-presented with worsening of the skin lesion.  [Excerpt from Cochrane Review [Dinnes J, Deeks JJ, Grainge MJ, Chuchu N, Ferrante di Ruffano L, Matin RN, Thomson DR, Wong KY, Aldridge RB, Abbott R, Fawzy M, Bayliss SE, Takwoingi Y, Davenport C, Godfrey K, Walter FM, Williams HC. Visual inspection for diagnosing cutaneous melanoma in adults. Cochrane Database of Systematic Reviews 2018, Issue 12. Art. No.: CD013194. DOI: 10.1002/14651858.CD013194. Accessed 29 March 2023.]:  “Reference standards  The ideal reference standard is histopathological diagnosis in all eligible lesions. A qualified pathologist or dermatopathologist should perform histopathology. Ideally, reporting should be standardised detailing a minimum dataset to include the histopathological features of melanoma to determine the American Joint Committee on Cancer (AJCC) Staging System (e.g. Slater 2014). We did not apply reporting of a minimum dataset as a necessary inclusion criterion, but extracted any pertinent information.  Partial verification (applying the reference test only to a subset of those undergoing the index test) was of concern given that lesion excision or biopsy are unlikely to be carried out for all benign‐appearing lesions within a representative population sample. Therefore, to reflect what happens in reality, we accepted clinical follow‐up of benign‐appearing lesions as an eligible reference standard, whilst recognising the risk of differential verification bias (as misclassification rates of histopathology and follow‐up will differ).  Additional eligible reference standards included cancer registry follow‐up and 'expert opinion' with no histology or clinical follow‐up. Cancer registry follow‐up is considered less desirable than active clinical follow‐up, as follow‐up is not carried out within the control of the study investigators. Furthermore, if participant‐based analyses as opposed to lesion‐based analyses are presented, it may be difficult to determine whether the detection of a malignant lesion during follow‐up is the same lesion that originally tested negative on the index test.  All of the above were considered eligible reference standards with the following caveats:   - all study participants with a final diagnosis of the target disorder must have a histological diagnosis, either subsequent to the application of the index test or after a period of clinical follow‐up; and - at least 50% of all participants with benign lesions must have either a histological diagnosis or clinical follow‐up to confirm benignity.” |

**Phase 2: Draw a flow diagram for the primary study**

| Figure 1. Post-referral pathway for DERM 2WW, 2-week wait; AIaMD, artificial intelligence-digital health technology.  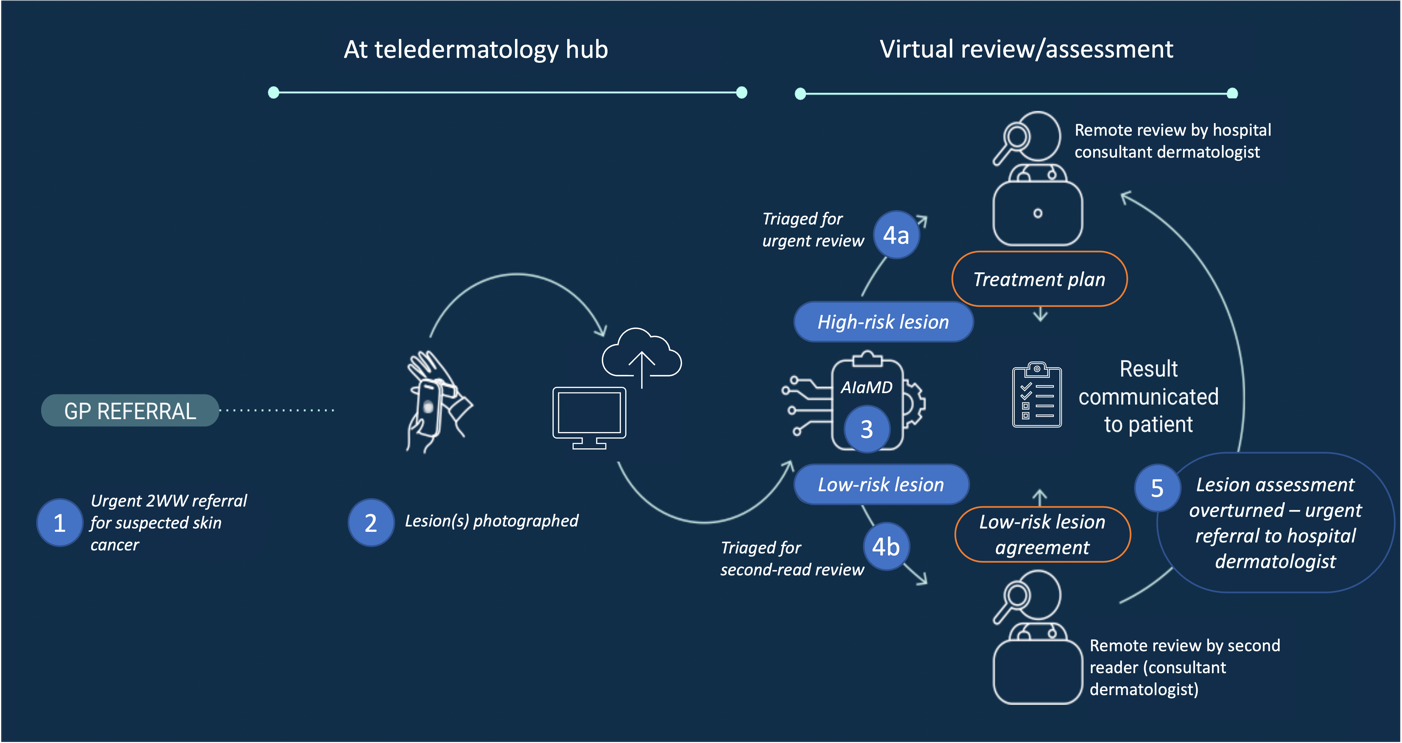 |
| --- |

**Phase 3: Risk of bias and applicability judgments**

*QUADAS-2 is structured so that 4 key domains are each rated in terms of the risk of bias and the concern regarding applicability to the research question (as defined above). Each key domain has a set of signalling questions to help reach the judgments regarding bias and applicability.*

**DOMAIN 1: PATIENT SELECTION**

**A. Risk of Bias**

| Describe methods of patient selection:  There was no selection, beyond application of eligibility criteria. The cases included in the analysis are accounted for within Figure 2 within the manuscript. All lesions from patients who had given additional consent, were assessed by DERM and had final diagnosis defined by histology for malignant lesions and by hospital dermatologist clinical assessment or histology if available for non-malignant lesions.  Comparison of the cases volumes seen by the SA pathway vs. the national cancer waiting times data demonstrated ~80% of all referrals to the trusts were seen within the pathway (see table below). |
| --- |

| SA cases vs CWT 2ww volumes | SA cases | CWT 2ww vol |  |
| --- | --- | --- | --- |
| UHB (Aug21-Sep22) | 11371 | 14410 | 78.9% |
| WSFT (Nov21-Sep22) | 2489 | 3162 | 78.7% |

❖ Was a consecutive or random sample of patients enrolled? ~~Yes/No/~~**Unclear**

❖ Was a case-control design avoided? **Yes**~~/No/Unclear~~

❖ Did the study avoid inappropriate exclusions? **Yes**~~/No/Unclear~~

**Could the selection of patients have introduced bias? RISK: ~~LOW/HIGH/~~UNCLEAR**

**B. Concerns regarding applicability**

| Describe included patients (prior testing, presentation, intended use of index test and setting)**:**  Patients are all those suspected of having a skin lesion being malignant based on GP examination in primary care. DERM would act as a triage tool for being seen by the hospital dermatologist.  All patients assessed with DERM from sites where there was information on biopsy or final clinical diagnosis (UHB and WSHT) and who had given consent. |
| --- |

**Is there concern that the included patients do not match the review question?**

**CONCERN: LOW/~~HIGH/UNCLEAR~~**

The population closely matches that where DERM would be used in practice.

**DOMAIN 2: INDEX TEST(S)**

**CONCERN: LOW/HIGH/UNCLEAR**

**If more than one index test was used, please complete for each test.**

**A. Risk of Bias**

| Describe the index test and how it was conducted and interpreted:  DERM is an AI system which analyses dermatoscopic images that were captured on a mobile phone by medical photographers and health care assistants. All patients who would normally be referred to hospital with lesions suspicious of being malignant are assessed by DERM if they are eligible (exclusions relate to patient age (>18) number (1-3) size, position and previous biopsy). DERM has a set in built fixed threshold. The algorithm was improved through techniques including additional training data and more granular lesion labels which was changed between version A & B. DERM produces a suspected diagnosis (melanoma, SCC, BCC, IEC, actinic keratosis, atypical naevus, or benign) and management outcome (discharge or refer to the Trust dermatologist for review) |
| --- |

❖ Were the index test results interpreted without knowledge of the results of the reference standard? Yes~~/No/Unclear~~

The time-line and process means that DERM cannot be influenced by the reference standard

❖ If a threshold was used, was it pre-specified? Yes~~/No/Unclear~~

**Could the conduct or interpretation of the index test have introduced bias? RISK: LOW/~~HIGH/UNCLEAR~~**

All the signal questions are yes.

**B. Concerns regarding applicability**

**Is there concern that the index test, its conduct, or interpretation differ from the review question?**

**CONCERN: LOW~~/HIGH/UNCLEAR~~**

The evaluation employs DERM exactly as it is intended to be used in practice

**DOMAIN 3: REFERENCE STANDARD**

**A. Risk of Bias**

| Describe the reference standard and how it was conducted and interpreted:  Where a biopsy is taken, the reference standard is based on that diagnosis. Where a biopsy is not taken the reference standard is based on the final diagnosis arrived at by the dermatologist. Where DERM triages the skin lesion as not requiring referral, the DERM images are checked by a second human reader who either confirms the discharge or re-refers the lesion back into the hospital system. These re-referrals and DERM referrals will initially be screened by a Trust dermatologist using the DERM images and any additional photographic images taken.  Representation to the hospital within 6 months is checked to confirm no worse or cancerous subsequent diagnosis of the same lesion after a previous discharge. |
| --- |

❖ Is the reference standard likely to correctly classify the target condition? Yes~~/No/Unclear~~

Although not perfect it does conform with reference standards specified in Cochrane reviews.

The use of a second read, probably means that the reference standard exceeds usual reference standards because all those triaged as non-referrals by the AI system go through a second read process and a high proportion of these referred for further assessment by the hospital dermatologist ahead of the final appointment in the hospital outpatients. This means that all AI negatives are seen by at least one clinician (looking at the AI images) , some by two clinicians (both looking at the AI images) and some three clinicians, the third of whom would have been the dermatologist at the out-patient appointment.

❖ Were the reference standard results interpreted without knowledge of the results of the index test? ~~Yes/No/~~Unclear

It is unclear whether the final diagnosis in the hospital outpatient clinic would have been influenced by the initial diagnosis of the AI. Currently as the technology is novel, we would expect clinicians to be sceptical of the value of the AI system. This may change in the future. Trust dermatologists would only review lesions identified as high risk by the AI or the second reviewer while second reviewer dermatologists are specifically in-role to look out for false negatives; therefore it is likely that there is higher vigilance and a greater threshold for discharging lesions as benign in this pathway.

**Could the reference standard, its conduct, or its interpretation have introduced bias? RISK: LOW~~/HIGH/UNCLEAR~~**

There is some lack of clarity about whether the reference standard could be influenced by the DERM assessment. Although this is possible, given current scepticism about AI in the clinical community, the judgement is that this possibility is unlikely to have introduced bias

**B. Concerns regarding applicability**

**Is there concern that the target condition as defined by the reference standard does not match the review question? CONCERN: LOW~~/HIGH/UNCLEAR~~**

The reference standard gives a good indication of the true disease state

**DOMAIN 4: FLOW AND TIMING**

**A. Risk of Bias**

| Describe any patients who did not receive the index test(s) and/or reference standard or who were excluded from the 2x2 table (refer to flow diagram):  See figure 2 in manuscript.  Describe the time interval and any interventions between index test(s) and reference standard:  Minimal delay where ref standard based on biopsy as patients are on a 2 week wait pathway. 6 months+ follow-up to confirm absence of re-presentation and subsequent worse diagnosis. |
| --- |

❖ Was there an appropriate interval between index test(s) and reference standard? Yes~~/No/Unclear~~

❖ Did all patients receive a reference standard? Yes~~/No/Unclear~~

❖ Did all patients receive the same reference standard? ~~Yes/~~No~~/Unclear~~

Differential verification bias is nearly always seen evaluations of the accuracy of skin cancer

❖ Were all patients included in the analysis? Yes~~/No/Unclear~~

**Could the patient flow have introduced bias? RISK: LOW~~/HIGH/UNCLEAR~~**

Although there is differential verification, and this might have introduced bias, the level of bias is similar to most other test accuracy evaluations in this disease area as they are also affected by differential verification
