# Supplementary material for: Real-world post-deployment performance of a novel machine learning-based digital health technology for skin lesion assessment and suggestions for post-market surveillance
Source: Front Med (Lausanne). 2023 Oct 31;10:1264846. doi: 10.3389/fmed.2023.1264846 (PMC10645139; doi:10.3389/fmed.2023.1264846)
Supplement: Supplementary file 2 [file Table_1.docx]

## Supplementary Table 1. Definitions for correct lesion classification and referral recommendation

BCC, basal cell carcinoma. FN, false negative; FP, false positive; SCC, squamous cell carcinoma; TN, true negative; TP, true positive.

**A. DERM classification true/false positive/negative for melanoma**

|  | **Final diagnosis** | |
| --- | --- | --- |
| **DERM Classification** | **Melanoma** | **Not  melanoma** |
| **Melanoma** | TP  (sensitivity) | FN |
| **Not melanoma** | FP | TN  (specificity) |

**B. DERM classification true/false positive/negative for malignancy (melanoma, SCC, BCC or rare skin cancer)**

|  | **Final diagnosis** | |
| --- | --- | --- |
| **DERM Classification** | **Malignancy** | **Not  malignancy** |
| **Malignancy** | TP  (sensitivity) | FN |
| **Not malignancy** | FP | TN  (specificity) |

**C. DERM referral recommendation**

|  | **Referral outcome** | |
| --- | --- | --- |
| **DERM recommendation** | **Refer** | **Do not  refer** |
| **Refer** | TP  (sensitivity) | FN |
| **Discharge*** | FP | TN  (specificity) |

*Discharge from the urgent two-week wait referral pathway.
