# Supplementary material for: Real-world post-deployment performance of a novel machine learning-based digital health technology for skin lesion assessment and suggestions for post-market surveillance
Source: Front Med (Lausanne). 2023 Oct 31;10:1264846. doi: 10.3389/fmed.2023.1264846 (PMC10645139; doi:10.3389/fmed.2023.1264846)
Supplement: Supplementary file 3 [file Table_2.docx]

## Supplementary Table 2. Details of repeat presentations to DERM

| **Repeat presentation case** | **Date*** | **DERM classification** | **Hospital dermatologist diagnosis** | **Management (decided by hospital dermatologist)** | **Histology diagnosis** |
| --- | --- | --- | --- | --- | --- |
| Case 1 | 2020-05-07 | Melanoma | Benign melanocytic nevus | Discharge | - |
|  | 2020-09-17 | Melanoma |  | Biopsy | Melanoma |
| Case 2 | 2020-06-09 | Excluded from DERM assessment: *‘Lesions which are subungual, or on mucosal, genital or palmoplantar surfaces’* | - | - | - |
|  | 2020-12-17 | Melanoma | Melanoma | F2F dermatology | Melanoma |
| Case 3 | 2020-06-29 | Excluded from DERM assessment: *‘Skin lesions too large to be entirely imaged within the dermoscopic device (≥15 mm)’* | [F2F] Ulcerated plaque | [F2F] F2F follow-up - missed two appointments | Not available as patient missed appointment |
|  | 2021-06-08 | Excluded from DERM assessment: *‘Open ulcerated skin lesions*  *and Skin lesions too large to be entirely imaged within the dermoscopic device (≥15 mm)’* | Other | F2F dermatology | BCC |
| Case 4 | 2020-09-29 | Melanoma | - | Punch biopsy | No biopsy taken, unknown as to why |
|  | 2021-05-26 | *Excluded from DERM assessment: ‘Open ulcerated skin lesions’* | SCC | Other | BCC |

*Dates are in year-month-day format.

BCC, basal cell carcinoma; F2F, face-to-face; IEC, intraepidermal carcinoma; SCC, squamous cell carcinoma
