# Supplementary material for: Real-world post-deployment performance of a novel machine learning-based digital health technology for skin lesion assessment and suggestions for post-market surveillance
Source: Front Med (Lausanne). 2023 Oct 31;10:1264846. doi: 10.3389/fmed.2023.1264846 (PMC10645139; doi:10.3389/fmed.2023.1264846)
Supplement: Supplementary file 4 [file Table_3.docx]

## Supplementary Table 3. Confusion matrices for post-deployment performance of DERM (lesion-level population)

The confusion matrices below show the final diagnosis defined by histology for malignant lesions and by dermatologist clinical assessment or histology if available for non-malignant lesions. The lesion-level analysis includes DERM-assessed lesions which had a final diagnosis and the patient provided additional consent for their data to be used for research and education.

| **A. DERM-vA (UHB)** | | | | | | | | |
| --- | --- | --- | --- | --- | --- | --- | --- | --- |
|  | **DERM Classification ⇔** | | | | | | | |
| **Final Diagnosis ⇓** | ***Melanoma*** | ***SCC*** | ***BCC*** | ***IEC*** | ***AK*** | ***Atypical*** | ***Benign*** | ***Total*** |
| **Melanoma** | 133 | 3 |  |  |  |  | 4 | 140 |
| **SCC** | 47 | 106 | 3 |  |  |  | 1 | 157 |
| **BCC** | 212 | 137 | 74 | 2 | 17 |  | 3 | 445 |
| **Malignant 'Other'** | 5 | 2 |  |  |  |  | 3 | 10 |
| **Bowen's disease (IEC)** | 92 | 102 | 4 | 5 | 17 |  | 10 | 230 |
| **Actinic Keratosis** | 222 | 173 | 11 | 4 | 93 |  | 34 | 537 |
| **Atypical Naevus** | 181 | 1 |  |  | 3 | 5 | 59 | 249 |
| **Refer 'Other'** | 13 |  |  |  |  |  | 3 | 16 |
| **Benign** | 1080 | 241 | 16 | 1 | 96 | 9 | 1408 | 2851 |
| **Total** | 1985 | 765 | 108 | 12 | 226 | 14 | 1525 | 4635 |

## B. DERM vA (WSFT)

|  | **DERM Classification ⇔** | | | | | | | |
| --- | --- | --- | --- | --- | --- | --- | --- | --- |
| **Final Diagnosis ⇓** | ***Melanoma*** | ***SCC*** | ***BCC*** | ***IEC*** | ***AK*** | ***Atypical*** | ***Benign*** | ***Total*** |
| **Melanoma** | 32 | 1 |  |  |  |  |  | 33 |
| **SCC** | 10 | 38 | 1 |  |  |  | 1 | 50 |
| **BCC** | 14 | 30 | 17 |  |  |  |  | 61 |
| **Malignant 'Other'** | 2 | 4 |  |  |  |  |  | 6 |
| **Bowen's disease (IEC)** | 8 | 13 |  | 3 |  |  | 3 | 27 |
| **Actinic Keratosis** | 31 | 53 | 8 |  | 6 |  | 1 | 99 |
| **Atypical Naevus** | 42 |  |  |  |  | 3 | 12 | 57 |
| **Refer 'Other'** |  |  |  |  |  |  |  | 0 |
| **Benign** | 142 | 70 | 7 |  | 3 | 1 | 153 | 376 |
| **Total** | 281 | 209 | 33 | 3 | 9 | 4 | 170 | 709 |

## C. DERM vB (UHB)

|  | **DERM Classification ⇔** | | | | | | | |
| --- | --- | --- | --- | --- | --- | --- | --- | --- |
| **Final Diagnosis ⇓** | ***Melanoma*** | ***SCC*** | ***BCC*** | ***IEC*** | ***AK*** | ***Atypical*** | ***Benign*** | ***Total*** |
| **Melanoma** | 58 |  |  |  |  |  |  | 58 |
| **SCC** | 5 | 40 |  |  |  |  |  | 45 |
| **BCC** | 15 | 41 | 18 | 2 |  |  |  | 76 |
| **Malignant 'Other'** |  | 1 |  |  |  |  |  | 1 |
| **Bowen's disease (IEC)** | 23 | 84 | 3 | 23 |  |  | 6 | 139 |
| **Actinic Keratosis** | 24 | 155 | 14 | 39 | 8 |  | 17 | 257 |
| **Atypical Naevus** | 115 |  |  |  |  | 4 | 74 | 193 |
| **Refer 'Other'** | 1 |  |  |  |  |  |  | 1 |
| **Benign** | 299 | 117 | 11 | 43 | 9 | 3 | 1333 | 1815 |
| **Total** | 540 | 438 | 46 | 107 | 17 | 7 | 1430 | 2585 |

## D. DERM vB (WSFT)

|  | **DERM Classification ⇔** | | | | | | | |
| --- | --- | --- | --- | --- | --- | --- | --- | --- |
| **Final Diagnosis ⇓** | ***Melanoma*** | ***SCC*** | ***BCC*** | ***IEC*** | ***AK*** | ***Atypical*** | ***Benign*** | ***Total*** |
| **Melanoma** | 18 |  |  |  |  |  |  | 18 |
| **SCC** | 1 | 12 | 1 |  |  |  |  | 14 |
| **BCC** | 4 | 13 | 17 |  |  |  |  | 34 |
| **Malignant 'Other'** | 1 | 1 |  |  |  |  |  | 2 |
| **Bowen's disease (IEC)** | 2 | 11 | 2 | 8 |  |  | 1 | 24 |
| **Actinic Keratosis** | 13 | 43 | 11 | 18 | 2 |  | 4 | 91 |
| **Atypical Naevus** | 39 |  |  |  |  | 5 | 21 | 65 |
| **Refer 'Other'** |  |  |  |  |  |  |  | 0 |
| **Benign** | 62 | 36 | 7 | 13 |  |  | 276 | 394 |
| **Total** | 140 | 116 | 38 | 39 | 2 | 5 | 302 | 642 |
